# Regression-Based Ranking of Pathogen Strains with Respect to Their Contribution to Natural Epidemics

**DOI:** 10.1371/journal.pone.0086591

**Published:** 2014-01-31

**Authors:** Samuel Soubeyrand, Charlotte Tollenaere, Emilie Haon-Lasportes, Anna-Liisa Laine

**Affiliations:** 1 UR546 Biostatistics and Spatial Processes, INRA, Avignon, France es that the sum of the estimated propor; 2 Metapopulation Research Group, Department of Biosciences, University of Helsinki, Helsinki, Finland; Memorial Sloan Kettering Cancer Center, United States of America

## Abstract

Genetic variation in pathogen populations may be an important factor driving heterogeneity in disease dynamics within their host populations. However, to date, we understand poorly how genetic diversity in diseases impact on epidemiological dynamics because data and tools required to answer this questions are lacking. Here, we combine pathogen genetic data with epidemiological monitoring of disease progression, and introduce a statistical exploratory method to investigate differences among pathogen strains in their performance in the field. The method exploits epidemiological data providing a measure of disease progress in time and space, and genetic data indicating the relative spatial patterns of the sampled pathogen strains. Applying this method allows to assign ranks to the pathogen strains with respect to their contributions to natural epidemics and to assess the significance of the ranking. This method was first tested on simulated data, including data obtained from an original, stochastic, multi-strain epidemic model. It was then applied to epidemiological and genetic data collected during one natural epidemic of powdery mildew occurring in its wild host population. Based on the simulation study, we conclude that the method can achieve its aim of ranking pathogen strains if the sampling effort is sufficient. For powdery mildew data, the method indicated that one of the sampled strains tends to have a higher fitness than the four other sampled strains, highlighting the importance of strain diversity for disease dynamics. Our approach allowing the comparison of pathogen strains in natural epidemic is complementary to the classical practice of using experimental infections in controlled conditions to estimate fitness of different pathogen strains. Our statistical tool, implemented in the R package StrainRanking, is mainly based on regression and does not rely on mechanistic assumptions on the pathogen dynamics. Thus, the method can be applied to a wide range of pathogens.

## Introduction

Development of epidemiological models has been driven by the need to understand and predict the dynamics, invasion, and persistence of plant and animal diseases [1]–[3]. The inherent variable nature of epidemics and heterogeneous spatial distribution of pathogens in their host populations has presented a challenge for this work [4], [5]. While variation in epidemics caused by abiotic environmental variation is relatively well understood [6], [7], quantifying the effect of intraspecific diversity in pathogen populations on epidemic rates has remained a challenge. This is non-trivial as diversity in traits affecting infection and transmission is a ubiquitous feature of pathogen populations [8].

Until recently, the scarcity of suitable genetic markers has impeded the study of variation in pathogen populations [9], [10]. However, with the development of Next Generation Sequencing, genetic tools are becoming increasingly available for parasites [11]–[13]. Molecular tracking of pathogen strains has the potential to identify disease transmission pathways across a variety of geographic scales [14]. At the very fine-scale of within host populations, molecular tools can reveal heterogeneities in transmission generated by differences in infectivity and subsequent growth and reproduction of different parasite strains [8], and their interactions with their hosts (genotype-by-genotype interactions; [15], [16]) and environment (genotype-by-environment interactions; [17], [18]).

Linking genetic pathogen data to epidemiological dynamics allows unraveling the role of pathogen intraspecific diversity in disease dynamics but this is currently limited by the availability of suitable analytical tools. Among the challenges to overcome in the development of these tools are: (i) The scarcity of data required to fit multi-strain dynamical models; (ii) The change of scale in the resolution from epidemiological data to the resolution of pathogen genetic data; and (iii) The ambiguity in the effect of the heterogeneity in pathogen strains, as discussed above. Here, we present an exploratory analysis tool based on data transformation, linear regression and kernel smoothing to assign ranks to different pathogen strains with respect to epidemiological spread, and we analyze whether this ranking is significant. The linear regression links epidemiological data (response variable) to genetic data (explanatory variables), and the kernel smoothing allows the scale of genetic data to be matched with the scale of epidemiological data. We apply our model to (i) data obtained under a statistical stochastic model, (ii) data obtained under a multi-strain mechanistic model (original model), and (iii) fine-scale within-season epidemiological data and genetical characterization of pathogen samples collected for the powdery mildew naturally infecting *Plantago lanceolata* in the archipelago of Finland. A recently developed protocol for field sampling of pathogen strains and Single Nucleotide Polymorphism (SNP) genotyping panel [19] allows a multilocus characterization of genetic content and subsequent distinction of different pathogen strains co-occurring within the same natural epidemic in this pathosystem.

## Materials and Methods

### Example of Field Data

#### Studied pathosystem

*Podosphaera plantaginis* (Castagne; U. Braun & S. Takamatsu) is a fungal pathogen specific to the ribwort plantain *Plantago lanceolata*. This species has been studied in the Åland archipelago (southwestern Finland), with long-term data evidencing metapopulation dynamics [20], [21]. *Po. plantaginis* belongs to the family of powdery mildews (Erysiphales, Ascomycete). These obligate pathogens develop conspicuous white-greyish mycelia on the surface of their host leaves and only penetrate the host tissue through feeding structures named haustoria. Continuous production of asexual spores named conidia leads to the succession of various overlapping asexual cycles during the summer (generation time varying between one and two weeks under controlled conditions). The powdery mildew population crashes during the winter due to a lack of living host tissue, but re-initiation of the epidemics in the following spring is enabled by the germination of sexual resting structures named chasmothecia.

#### Epidemiological data

Small-scale data were collected during the summer 2011 within one *Pl. lanceolata* meadow (ID 609) located in the Eckero part of the Åland archipelago (Neither the host plant nor the pathogen is protected species and Finnish legislation (Jokamiehenoikeus) allows the sampling of wild species to everyone). This *Pl. lanceolata* population (approx. 2400 m^2^) was visited weekly between July 18 and August 25 (week 29 to 34), except on week 33 (five observations in total). Pattern of infection within host populations is known to be highly aggregated in this pathosystem [22]. Consequently, the survey was performed by dividing the studied location into 122 squared grid cells of 9 m^2^. Every week, each cell was visually inspected, and the number of host leaves infected by the powdery mildew was recorded.

#### Genetic data

On the last day of the survey (last week of August), 45 infected leaves were sampled for genetic characterization. Samples were chosen from the different infected cells so that, in each cell, the number of collected samples was approximately related to the disease intensity. Genotyping of the fungal pathogen for 27 SNP markers was performed as described in [19]. This methodology consists of direct genotyping (no purification step) of the total fungal material found on one infected leaf and allows to detect whether infection of the leaf was caused by a unique vs multiple fungal strains [19]. Clear multilocus data obtained from unique infections were used to define the different fungal strains circulating within the population and to attribute each sample to a particular fungal strain. Most of the mixed-genotype infections could be assigned to a mix of two identified strains whereas few remained unattributed and were removed from the dataset.

### Regression Model for the Analysis of Strain Contributions

Consider a host population covering a spatial domain divided into 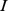 similar grid cells. The cells are labelled by 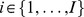. Two types of observations are made: epidemiological observations and pathogen genetic observations. The epidemiological data are pathogen intensities 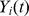 in cells 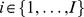 at times 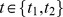. The pathogen genetic data observed at time 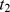 are 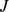 samples randomly collected in the grid cells and classified into a set of 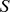 strains. The label 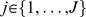 is used to identify the samples. The label 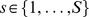 is used to identify the strains. For each sample 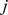, 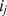 is the cell where 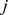 was collected and 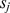 is the strain of 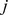, and 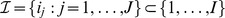 is the set of cells containing genetic samples. We introduce the variable 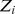 satisfying:
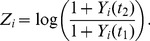
(1)



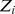
 is built to characterize the growth of the epidemic in cell 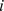 between times 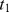 and 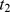 (the growth can be negative; see Discussion for other 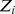
’s constructions). We assume that 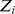 depends on the strains that are locally present at time 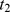 in the following way:
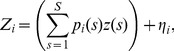
(2)where 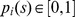 is the proportion of strain 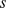 in cell 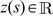, 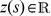 is the intrinsic growth rate of strain 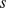 (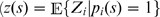 is the expectation of 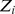 if only strain 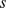 is in cell 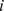), and 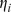 is a centered normal random noise (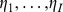 are assumed to be independent).

The model (2) can be viewed as a regression linear model where 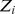 is the response variable, 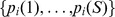 is the vector of explanatory variables and 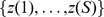 are the regression coefficients. Ranking the pathogen strains with respect to their contributions to field epidemics is achieved by ranking the coefficients 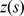, 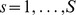.

### Approximation of the Regression Model

In model (2), the explanatory variables 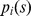 (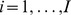, 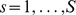) are not observed. However, using the pathogen genetic data and a kernel smoothing technique, the proportions 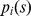 can be estimated and plugged in Equation (2). Let 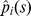 be an unbiased estimate of 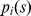, i.e. 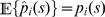, we replace the model (2) by the following approximate regression model:
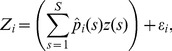
(3)where 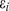 is a centered normal random noise with variance 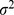 (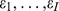 are assumed to be independent). The term 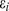 is a noisy version of 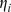 because of the difference between 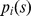 and 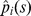. We ignore the eventual dependence in the 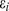 to keep the model simple (since it is only used like an exploratory tool) but such a dependence could be taken into account; see Discussion. In the model (3), we used the following weighted estimate of 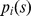:
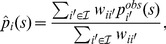
(4)where 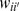 is the weight of pathogen samples collected in cell 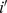 to estimate the proportion of strain 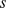 in cell 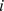, 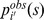 is the observed proportion of strains 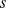 in cell 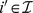,



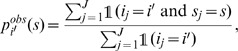
and 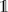 is the indicator function 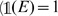 if event 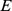 holds, zero otherwise). The denominator in Equation (4) ensures that the sum of the estimated proportions 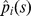 over 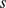 is equal to one. We used a kernel form for 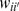 to give positive weights to samples collected in neighbor cells:
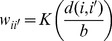
(5)where 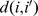 is the distance between the centers of cells 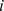 and 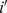, 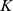 is the quadratic kernel 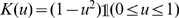, and 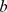 is a bandwidth determining the extent of the kernel and, consequently, the number of observed proportions 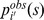 used to estimate each true proportion 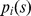. By convention, under the case 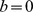, 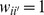 if 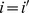 and zero otherwise.

In kernel smoothing, the choice of the bandwidth 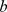 corresponds to a trade-off between bias and variance of the estimates [23], [24]. Smaller the bandwidth, smaller the bias and larger the variance. Larger the bandwidth, larger the bias and smaller the variance. In the context that we are considering here, a too small bandwidth with respect to the amount of information available in the data may lead to strongly varying estimates of the strain proportions and, therefore, to state that a difference in the parameters 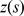 is significant whereas it is not. Conversely, when the bandwidth is increased, the estimated proportions of strains tend to be homogeneous in space and, therefore, we will not be able to detect any differences between the parameters 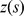. Testing the method on simulations will help us in assessing the effect of the bandwidth 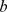 for a given amount of information.

### Ranking of Pathogen Strains

We use the approximate regression model (3) to rank the pathogen strains in their contributions to natural epidemics. In this model, 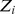 is the response variable, 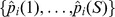 is the vector of explanatory variables and 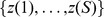 are the regression coefficients. A simple linear regression is then carried out (e.g. using the *lm()* function of the R statistical software) to obtain point estimates 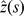 of the coefficients 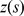, 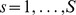. Then, the coefficients 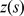 can be ranked by using their estimated values.

To assess whether the ranking in the coefficients 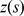 is significant, we adopted a permutation approach [25]. For 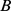 different independent random permutations, say 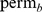 (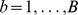), of the indices 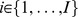 for which the proportions 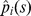 can be computed, we estimated the coefficients 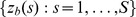 of the following model:
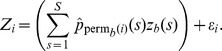



Let 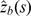 denote the estimate of 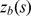 obtained by fitting the previous model. Then, for two strains 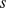 and 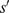 (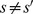), the 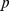-value of the unilateral difference permutation test 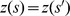 versus 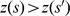 is: 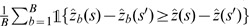


Because several pairwise tests can be carried out when there are more than two strains, the usual significance level 0.05 for a 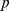-value is too high [26]. The simple Bonferroni’s correction that consists of dividing the significance level by the number of tests should be very conservative for the null hypothesis because the ranking tests are dependent. However, we will apply this correction and test it on simulations.

### Test Data Sets Obtained Under the Regression Model

Our method consists of performing a linear regression with noisy explanatory variables. Thus, the convergence results of classical linear regression do not hold [27]. To assess the effect of using noisy explanatory variables, namely the estimated proportions 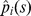, we applied the method to simulated data sets obtained under the regression model (2) but analyzed with the model (3). The technical details and the detailed results are provided in File S1 (Sec. A).

### Test Data Sets Obtained Under a Mechanistic Model

In practice, our method will be applied to data *generated* under mechanistic processes that are more complex than model (2). To assess the effect of using a regression model for the analysis instead of the true mechanistic model, we applied the method to simulated data sets obtained under an original mechanistic model detailed in File S1 (Sec. B). In this model, the epidemic spreads over a 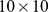 square grid with inter-node distance equal to one (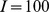), and at discrete integer times 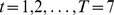. The epidemic is the sum of 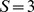 sub-epidemics corresponding to 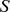 strains. The 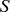 sub-epidemics are mutually independent. Each sub-epidemic is randomly initiated in time and in intensity. The growth and the spread of the sub-epidemics are governed by Poisson distributions and an exponential dispersal kernel. The spread depends on a dispersal parameter 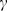 that is the same for the 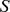 strains. The growth depends on a coefficient 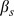 that represents the fitness of strain 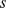. The coefficients 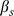 in the mechanistic model are the counterparts of the coefficient 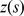 in the regression model (2).

We carried out 1,600 simulations of the mechanistic model; 800 with the dispersal parameter 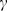 equal to 0.2 (short dispersal distances; see illustration in File S1, Sec. B, Figures S4 and S6), 800 with 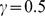 (longer dispersal distances; see illustration in File S1, Sec. B, Figure S8). Among each series of 800 simulations, 200 were made with equal coefficients: 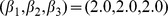, 200 with slight differences in the coefficients: 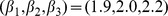, 200 with intermediate differences: 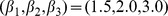, and 200 with large differences: 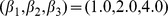. To study the effect of the sampling effort, different sample sizes were considered for the genetic data: we used different numbers of sampling sites (10, 20 and 30) and different numbers of samples per sampling site (1, 5 and 10); see details in File S1 (Sec. B). For each simulation and each sampling effort, we tested the hypothesis of no difference in the coefficients for each pair of strains (1 and 2; 2 and 3; 1 and 3) by using the unilateral permutation test orientated with respect to the estimated coefficients (e.g. if 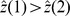, we tested 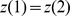 versus 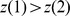). Then, in each case, we counted the numbers of adequate and inadequate rejections of the null hypothesis among 200 repetitions.

### Computer Code

An R package entitled StrainRanking containing the ranking method, the real data and generators of data under the regression and mechanistic models is available in the CRAN package repository. In this package, the ranking is carried out with the function entitled ranking.strains().

## Results

### Application to Simulations

The application of the method to simulations performed under the regression model is shown in File S1 (Sec. A; Figures S1, S2, S3; Tables S1, S2). The following general conclusions can be drawn. (i) One rarely rejects the null hypothesis for the wrong alternative hypothesis. (ii) The larger differences between the coefficients 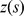 are more often detected than the smaller ones. (iii) Increasing the bandwidth leads to a more powerful test but slightly increases the number of times that the wrong alternative is accepted. (iv) More importantly, the ranking method is efficient despite the use of noisy explanatory variables.

Then, the method was applied to simulations under the mechanistic model. In this case, the model used to analyze the data is definitely different from the model used to simulate the data, but, with our ranking method, we expect to detect a signature of the variation in strain fitness (the signature is the ranking). The contributions of the pathogen strains to the epidemics are measured with the coefficients 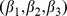 in the mechanistic *simulation* model, and with the coefficients 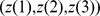 in the regression *analysis* model. The rankings of 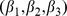 and 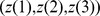 should be the same. Here, we consider two values (0.2 and 0.5) of the dispersal parameter 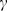 to see in which situation the method is able to detect the variation in strain fitness.

**Figure 1:**
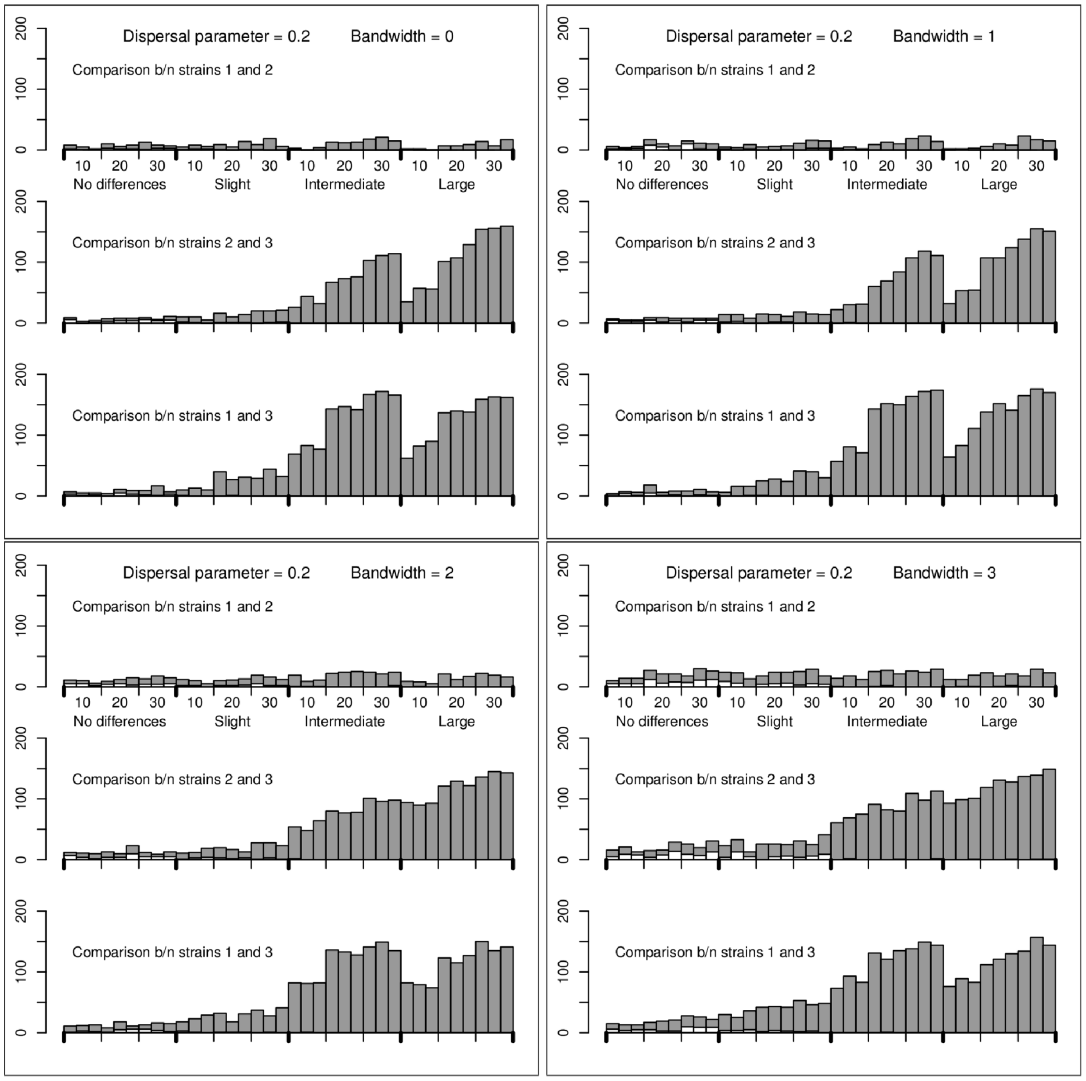
Numbers of test rejections for simulations performed under the mechanistic model with dispersal parameter *γ* = 0.2. Grey bars: number of times that the null hypothesis was rejected and that the alternative was true; White bars: number of times that the null hypothesis was rejected and that the alternative was wrong. The rejection threshold was fixed at 0.05/3 (using Bonferroni’s correction). The number of sampling sites and the differences in the fitness coefficients are given under the x-axis of the top panels. Moreover, between each consecutive ticks, there are three bars corresponding, from left to right, to 1, 5 and 10 collected samples per sampling site. The results are provided for the bandwidth values 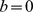 (top left), 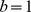 (top right), 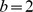 (bottom left) and 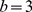 (bottom right).

**Figure 2:**
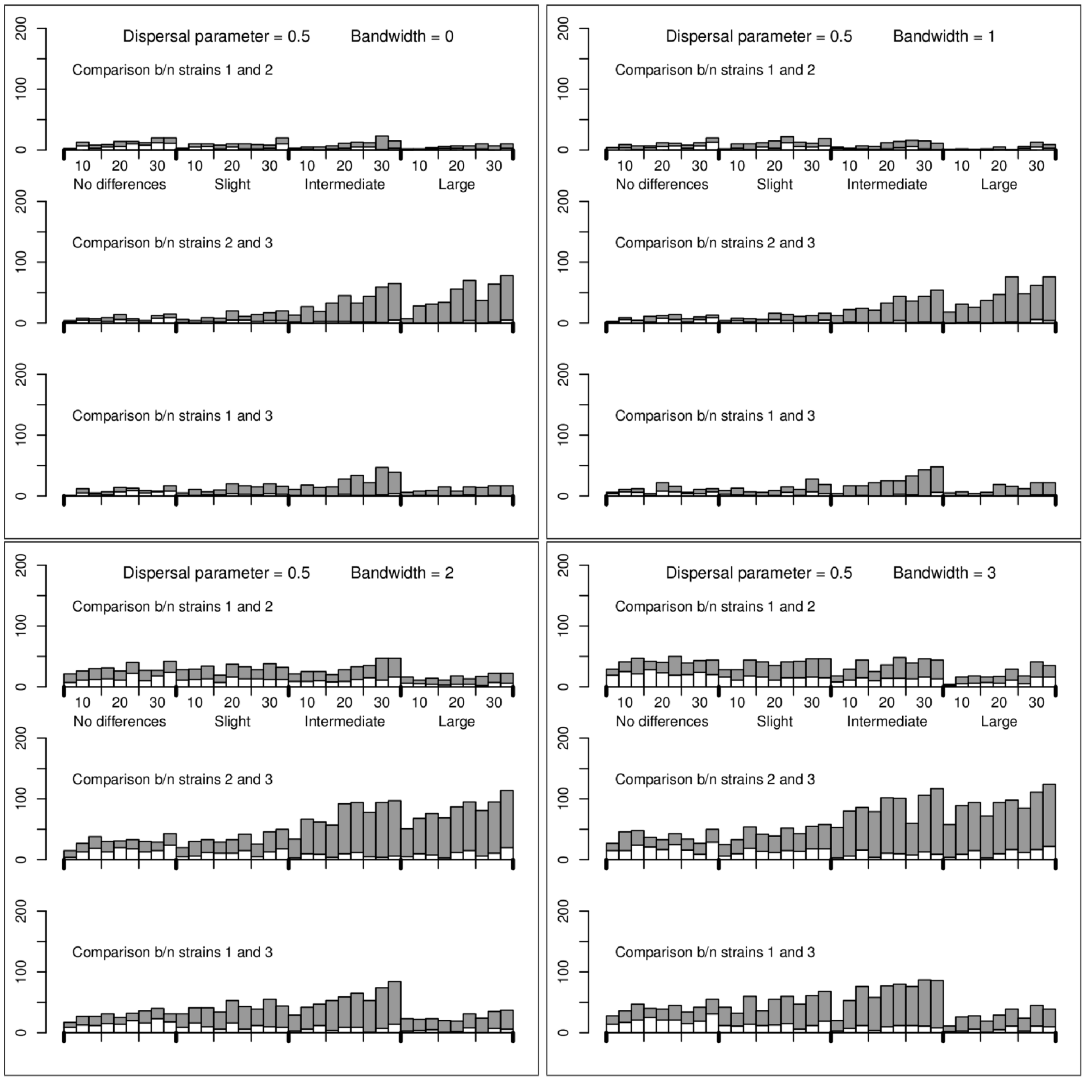
Numbers of test rejections for simulations performed under the mechanistic model with dispersal parameter *γ* = 0.5. Grey bars: number of times that the null hypothesis was rejected and that the alternative was true; White bars: number of times that the null hypothesis was rejected and that the alternative was wrong. The rejection threshold was fixed at 0.05/3 (using Bonferroni’s correction). The number of sampling sites and the differences in the fitness coefficients are given under the x-axis of the top panels. Moreover, between each consecutive ticks, there are three bars corresponding, from left to right, to 1, 5 and 10 collected samples per sampling site. The results are provided for the bandwidth values 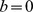 (top left), 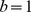 (top right), 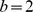 (bottom left) and 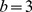 (bottom right).

The application of the method to three simulations performed under the mechanistic model (with equal or different coefficients 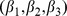 and with two different values of 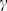) is detailed in File S1 (Sec. B; Figures S4, S5, S6, S7, S8, S9; Tables S3, S4, S5) where examples of simulated multi-strain dynamics are also displayed. Here, we only provide the results obtained for the series of simulations. For each bandwidth 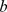 (0, 1, 2, 3), each sampling effort and each dispersal parameter 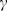 (0.2, 0.5), Figures 1 and 2 show the numbers of times among 200 repetitions that the null hypothesis (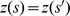) was rejected. The rejection threshold was fixed at 0.05/3, using Bonferroni’s correction.

The following conclusions can be drawn. When the dispersal parameter 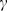 is 0.2 (short dispersal), the conclusions are similar to those drawn with the regression model: (i) one rarely rejects the null hypothesis for the wrong alternative hypothesis (white bars); (ii) the larger differences between the coefficients 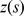 are more often detected than the smaller ones; (iii) increasing the bandwidth slightly increases the number of times that the wrong alternative is accepted. When the dispersal parameter is 0.5 (long dispersal), the test is less powerful and increasing the bandwidth increases the risk of accepting the wrong alternative hypothesis. Besides, when the differences between the strains are large, the power to detect differences between strain 1 (the less fit) and the other strains is very low. The reason is that, most often, strain 1 has a relatively negligible intensity and is not sampled.

### Application to Powdery Mildew of *Plantago lanceolata*

**Figure 3:**
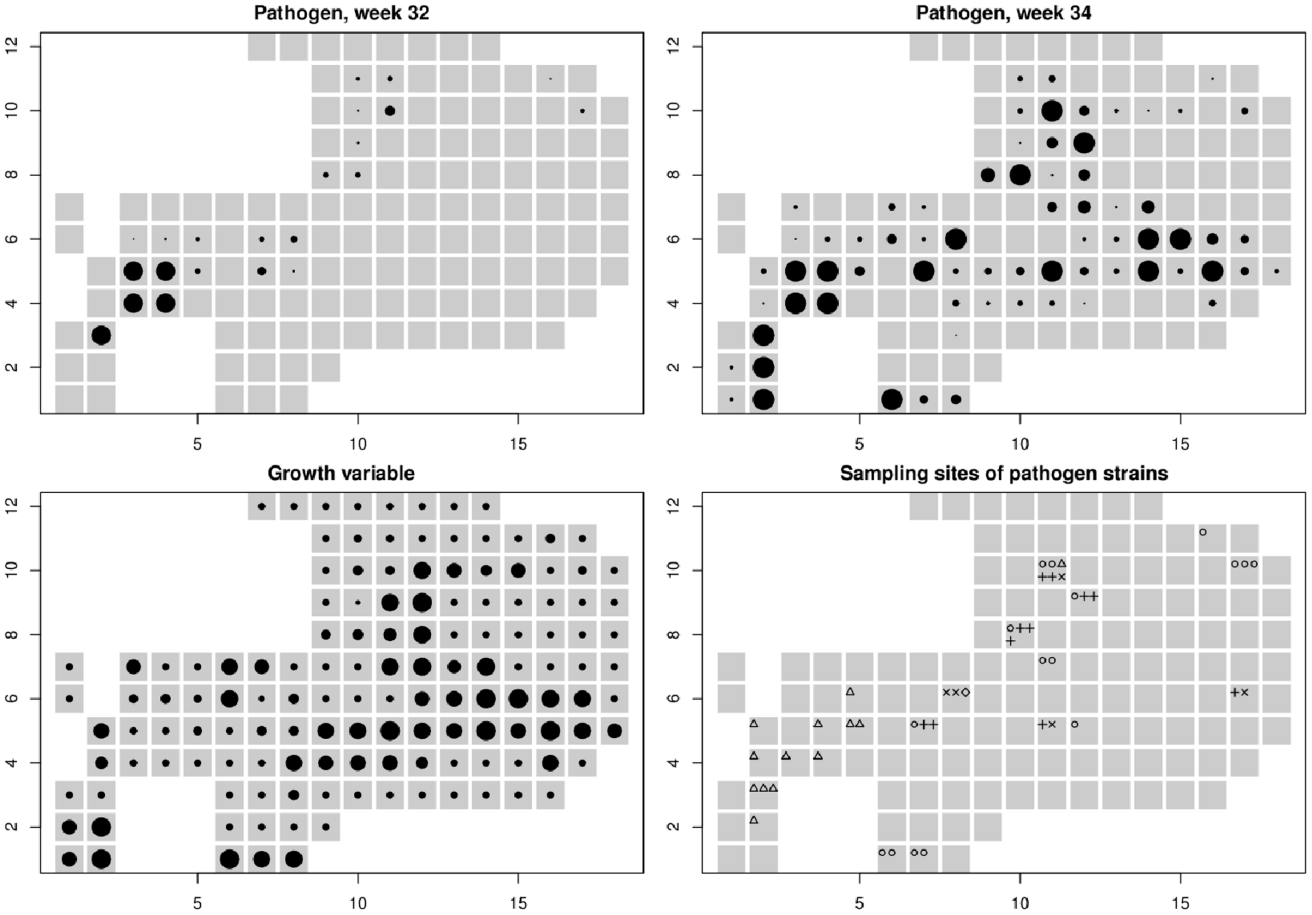
Powdery mildew data and growth variable in a patch of *Plantago lanceolata* in the Åland islands. In all the panels the grey squares represent the grid cells covering the host population. Top panels: number of infected leaves in 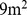 square cells at weeks 32 (left) and 34 (right) of year 2011 (dot size proportional to number of infected leaves that ranges between 0 and 137). The axis scales indicate the number of the cell from the most bottom-left cell. Bottom left: Growth variable 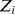 (dot size linear in 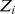 that ranges between −1.01 and 4.93). Bottom right: sites where samples were collected (46 samples in 22 sites; diamond: strain 1; plus: strain 2; triangle: strain 3; circle: strain 4; cross: strain 5).

The ranking method was applied to the field data of powdery mildew epidemics displayed in Figure 3. Among the 44 collected samples, 40 represented single-strain infections, leading to the identification of five different strains within the pathogen population. Among the four mixed-genotype infections, three could be attributed to a mix of previously identified strains, whereas one remained unattributed and was removed from the dataset. For the analysis, we used the intermediate bandwidth 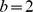 to decrease the risk of detecting false positive differences. In addition, since there are five strains, the total number of unilateral tests is 10 and the rejection threshold is of the order 0.05/10, using Bonferroni’s correction. However, this threshold value is certainly very conservative (tendency to under-reject the null hypothesis of coefficient equality when this hypothesis is wrong).

**Figure 4:**
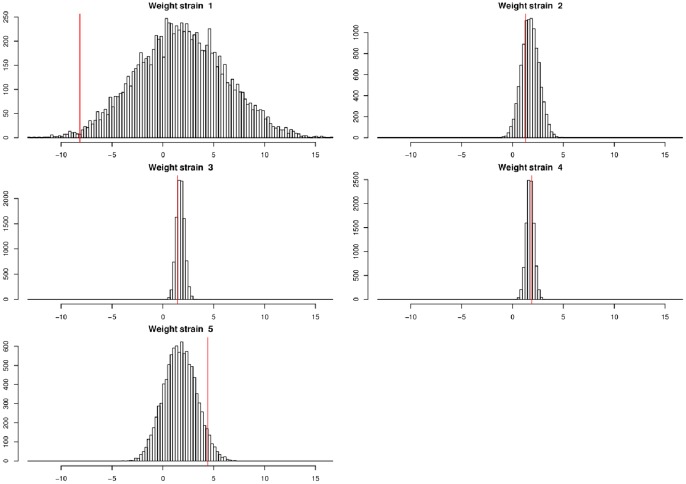
Estimated values of the coefficients *z*(*s*) for powdery mildew strains collected in a patch of *Plantago lanceolata* (vertical lines) and corresponding permutation-based distributions obtained under the null hypothesis of coefficient equality (histograms).

Table 1 provides the number of samples per strain, the estimated values of the coefficients 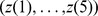 and the *p*-values associated with the unilateral permutation tests. Figure 4 shows the estimated values of the coefficients and their permutation-based distributions under the null hypothesis of coefficient equality. Based on the available data, there is no significant difference in strain fitness at the (certainly too conservative) rejection threshold 0.005 (the relatively low *p*-values concerning strain 1 have to be considered with caution since there is only one sample corresponding to this strain). Nevertheless, strain 5 tends to have a higher fitness than the other strains and, to be able to conclude in future studies about differences in strain fitness, more genetic samples should be gathered.

**Table 1:** Results of the ranking of pathogen strains observed within a natural epidemic of powdery mildew in *Plantago lanceolata*.

Strain	1	2	3	4	5
Number of samples	1	11	13	16	5
	*z*(1)	*z*(2)	*z*(3)	*z*(4)	*z*(5)
Estimated value	−8.16	1.29	1.43	1.88	4.42
		*z*(2)>*z*(1)	*z*(3)>*z*(1)	*z*(4)>*z*(1)	*z*(5)>*z*(1)
*p*-value		0.007	0.010	0.008	0.011
			*z*(3)>*z*(2)	*z*(4)>*z*(2)	*z*(5)>*z*(2)
*p*-value			0.446	0.294	0.078
				*z*(4)>*z*(3)	*z*(5)>*z*(3)
*p*-value				0.238	0.038
					*z*(5)>*z*(4)
*p*-value					0.064

The table provides frequencies of strains in the whole sample, estimated values of coefficients 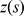, and test *p*-values indicating the significance of the ranking between the pathogen strains. The strains were ordered with respect to the estimated values of 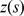.

## Discussion

The method presented here was developed to assign ranks to different pathogen strains with respect to their contribution to natural epidemics. As shown with a simulation-based study, the method can achieve this aim. Importantly, the success of the method depends on the design of the field survey as the statistical power of our approach depends on the sampling size (larger the sample, better the detection of actual differences between strains) and on the sampling scale (large dispersal distances with respect to the spatial extent of the sampling may decrease the power of our approach).

To reach more accurate ranking, the method could be improved as follows. (i) Other growth variables 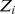 could lead to a larger statistical power and robustness. We could especially use multidimensional growth variables to handle different features of pathogen spread. We could also build growth variables that depend on the host population to take into account an eventual limiting capacity due to low host densities. (ii) A spatially dependent heteroscedastic and non-stationary noise could replace the white noise in the analysis regression model to take into account the dependence between neighbor cells due to the dispersal of the pathogen. A residual analysis like in [28] and [29] could be carried out to specify the noise structure. (iii) If the observed proportions 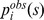 of pathogen strains are not based on the same number of pathogen samples, then the weights 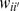 could be modified to avoid to give strong weights to strongly uncertain observed proportions. (iv) Finally, an automatic selection of the bandwidth *b* and the rejection threshold could be developed using cross-validation or Monte Carlo simulations performed under the fitted regression model.

Disease ecologists commonly use experimental infections in controlled conditions to estimate the fitness of different pathogen strains/genotypes (see for example [17], [30]–[36]). But how the differences between strains found in lab-measured fitness translate to the actual performance in the field has never been assessed to our knowledge. In this study, we developed analytical tools to estimate field performance of pathogen genotypes, allowing further comparison with fitness measures of pathogen strains estimated under controlled conditions. Simple correlations between the two measures for each pathogen genotype may however not be expected if the pathogen genotype interacts with local host genotypes, or with the local environment, to determine the pathogen’s fitness [17]. However, such comparisons are useful for estimating how complex the experimental design needs to be if aiming at predicting the pathogen performance under natural conditions.

Our statistical exploratory tool mainly based on regression does not rely on mechanistic assumptions on the pathogen dynamics. Therefore, it can be applied to a wide range of pathogens for which epidemiological and genetic data can be collected during natural epidemics or during experimental epidemics in crop fields. To facilitate the application of the method to other pathogens, an open source computer code is available. The code can be modified and extended by the user to meet the user’s requirements.

## Supporting Information

File S1 **Supporting information providing methodological details and complementary results.**
(PDF)
