# Supplementary material for: Regression-Based Ranking of Pathogen Strains with Respect to Their Contribution to Natural Epidemics
Source: PLoS One. 2014 Jan 31;9(1):e86591. doi: 10.1371/journal.pone.0086591 (PMC3909007; doi:10.1371/journal.pone.0086591)
Supplement: File S1 — Supporting information providing methodological details and complementary results. (PDF) [file pone.0086591.s001.pdf]

Supporting Information

Regression-based ranking of pathogen strains with respect to their contributions to natural epidemics

S. Soubeyrand[*,†], C. Tollenaere[‡,§], E. Haon-Lasportes[*], and A.-L. Laine[‡]

A Simulation under the regression model and results

A.1 Simulation model and tests

We considered a 10×10 square grid with inter-node distance equal to one. In each grid cell, the proportions of three strains were drawn from independent Dirichlet distributions with spatially varying means and variances defined using sine and cosine functions applied to the coordinates of the cells:

$$(p_i(1), p_i(2), p_i(3)) \sim \text{Dirichlet}[100\{\cos(x_{2,i}) + 1.5, \sin(x_{1,i}) + 1.5, \sin(x_{2,i}) + 1.5\}], \qquad \text{(S1)}$$

where$(x_{1,i}, x_{2,i})$ are the coordinates of cell center i. The growth variables Z_i were generated by simulating the normal variables η_i and applying the formula defining Z_i (Equation (2) in the main text):

$$Z_i = \left(\sum_{s=1}^{3} p_i(s) z(s) \right) + \eta_i.$$

We carried out 800 simulations of the true model, 200 with equal coefficients: $(z(1), z(2), z(3)) = (2.0, 2.0, 2.0)$, 200 with slight differences in the coefficients: $(z(1), z(2), z(3)) = (1.9, 2.0, 2.2)$, 200 with intermediate differences: $(z(1), z(2), z(3)) = (1.5, 2.0, 3.0)$, and 200 with large differences: $(z(1), z(2), z(3)) = (1.0, 2.0, 4.0)$. For each simulation, n sampling sites were randomly and uniformly drawn among the 100 grid cells. Then, multinomial distributions of size m and with probabilities given by Equation (S1) were drawn to generate the pathogen samples

[*]INRA, UR546 Biostatistics and Spatial Processes, 84914 France
[†]E-mail: Samuel.Soubeyrand@avignon.inra.fr
[‡]Metapopulation Research Group, Department of Biosciences, P.O. Box 65 (Viikinkaari 1), 00014 University of Helsinki, Helsinki, Finland
[§]Present address: IRD, UMR Résistance des Plantes aux Bioagresseurs (IRD-CIRAD-UM2). 911, Avenue Agropolis. BP 64501 F- 34394 MONTPELLIER Cedex 5 France.

($J = n \times m$). We used different numbers of sampling sites ($n \in \{10, 20, 30\}$) and different numbers of samples per sampling site ($m \in \{1, 5, 10\}$) to study the effect of the sampling effort. Regarding the bandwidth, we tested four different values: $b \in \{0, 1, 2, 3\}$. By doing so, we took into account up to 28 neighbor cells in the estimation of $p_i(s)$.
For each simulation and each sampling effort, we tested the hypothesis of no difference in the coefficients for each pair of strains (1 and 2; 2 and 3; 1 and 3) by using the unilateral permutation test orientated with respect to the estimated coefficients (e.g. if $\hat{z}(1) > \hat{z}(2)$, we tested $z(1) = z(2)$ versus $z(1) > z(2)$). Consequently, in each case, we can count the numbers of adequate and inadequate rejections of the null hypothesis among 200 repetitions.

A.2 Application to a simulation with equal coefficients

We considered one of the simulations described above corresponding to $z(1) = z(2) = z(3) = 2$. We used 30 sampling sites and 10 samples per site. Strain proportions were estimated with $b = 1$. Figure S1 and Table S1 show the simulation and provide the results. In this case, the ranking was not significant.

Table S1: Results of the unilateral tests obtained for a simulation under the regression model with equal coefficients ($z(1) = z(2) = z(3) = 2$).

	$z(1)$	$z(2)$	$z(3)$
True value	2.00	2.00	2.00
Estimated value	2.06	1.94	2.02
	$z(1) > z(2)$	$z(1) > z(3)$	$z(3) > z(2)$
p-value	0.209	0.384	0.241

A.3 Application to a simulation with increasing coefficients

We considered one of the simulations described above corresponding to $z(1) = 1.5$, $z(2) = 2$ and $z(3) = 3$. We used 30 sampling sites and 10 samples per site. Strain proportions were estimated with $b = 1$. Figure S2 and Table S2 show the simulation and provide the results. In this case, the ranking between strains 3 and 1 and between strains 3 and 2 was correct and significant. The ranking between strains 2 and 1 was correct but not significant.

A.4 Application to series of simulations

For each combination of the bandwidth b (0, 1, 2 or 3), the number n of sampling sites (10, 20 or 30) and the number m of collected strains per sampling point (1, 5 or 10), Figure S3 shows the numbers of times among 200 repetitions that the null hypothesis ($z(s) = z(s')$) was rejected. The rejection threshold was fixed at 0.05/3 (using Bonferroni's correction). The following conclusions can be drawn. One rarely reject the null hypothesis for the wrong alternative hypothesis (white bars). The larger differences between the coefficients

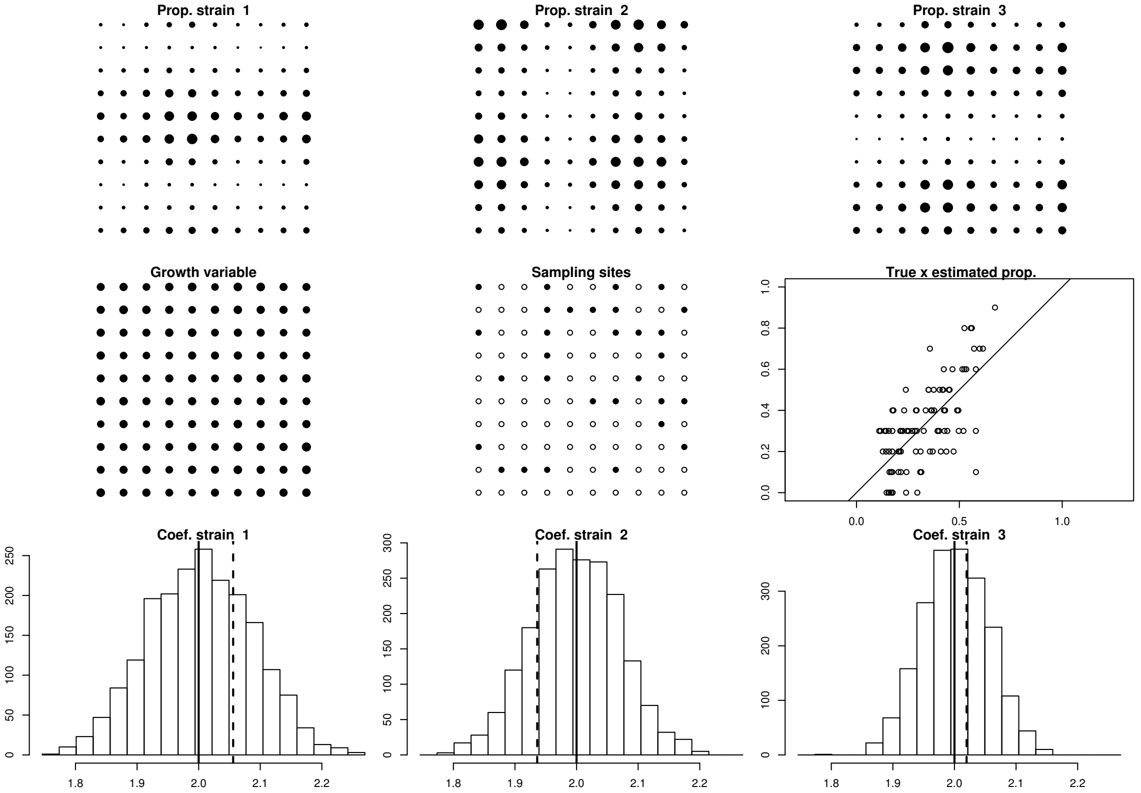

Figure S1: Data and results obtained for a simulation under the regression model with equal coefficients ($z(1) = z(2) = z(3) = 2$). The space is a 10×10 square grid. Top panels: Simulated proportions of each strain. Centre-left: Simulated values of Z_i (growth variable). Centre: Sampling sites (filled circles). Centre-right: True values of proportions of strains (abscissa) versus estimated values (ordinate). Bottom panels: True (solid vertical lines) and estimated (dashed vertical lines) values of the coefficients $z(s)$, and corresponding permutation-based distributions under the null hypothesis of coefficient equality (histograms).

$z(s)$ are more often detected than the smaller ones. Increasing the bandwidth leads to a more powerful test (in particular for the smallest differences in the $z(s)$ –between strains 1 and 2– and when the number of collected strains per sampling sites is low) but slightly increase the number of times that the wrong alternative is accepted.

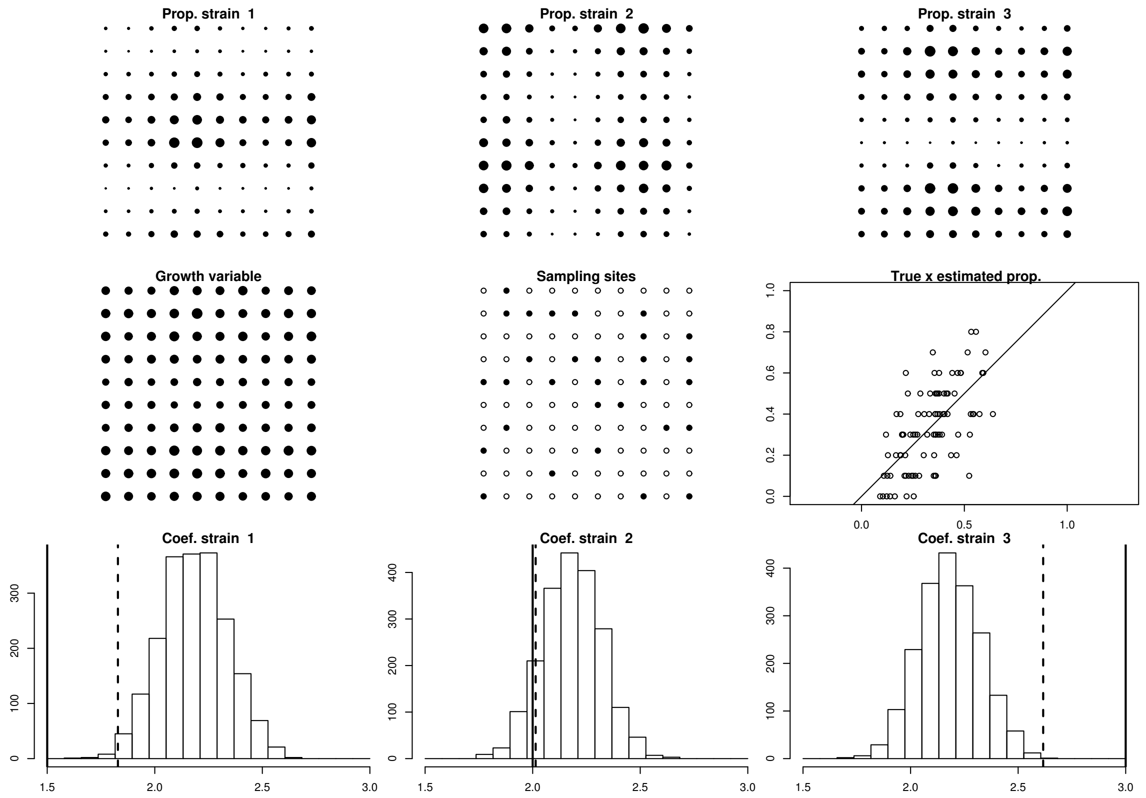

Figure S2: Data and results obtained for a simulation under the regression model with increasing coefficients ($z(1) = 1.5$, $z(2) = 2$ and $z(3) = 3$). The space is a 10×10 square grid. Top panels: Simulated proportions of each strain. Centre-left: Simulated values of Z_i (growth variable). Centre: Sampling sites (filled circles). Centre-right: True values of proportions of strains (abscissa) versus estimated values (ordinate). Bottom panels: True (solid vertical lines) and estimated (dashed vertical lines) values of the coefficients $z(s)$, and corresponding permutation-based distributions under the null hypothesis of coefficient equality (histograms).

Table S2: Results of the unilateral tests obtained for a simulation under the regression model with increasing coefficients ($z(1) = 1.5$, $z(2) = 2$ and $z(3) = 3$).

	$z(1)$	$z(2)$	$z(3)$
True value	1.50	2.00	3.00
Estimated value	1.83	2.01	2.62
	$z(2) > z(1)$	$z(3) > z(1)$	$z(3) > z(2)$
p-value	0.224	5e-04	0.008

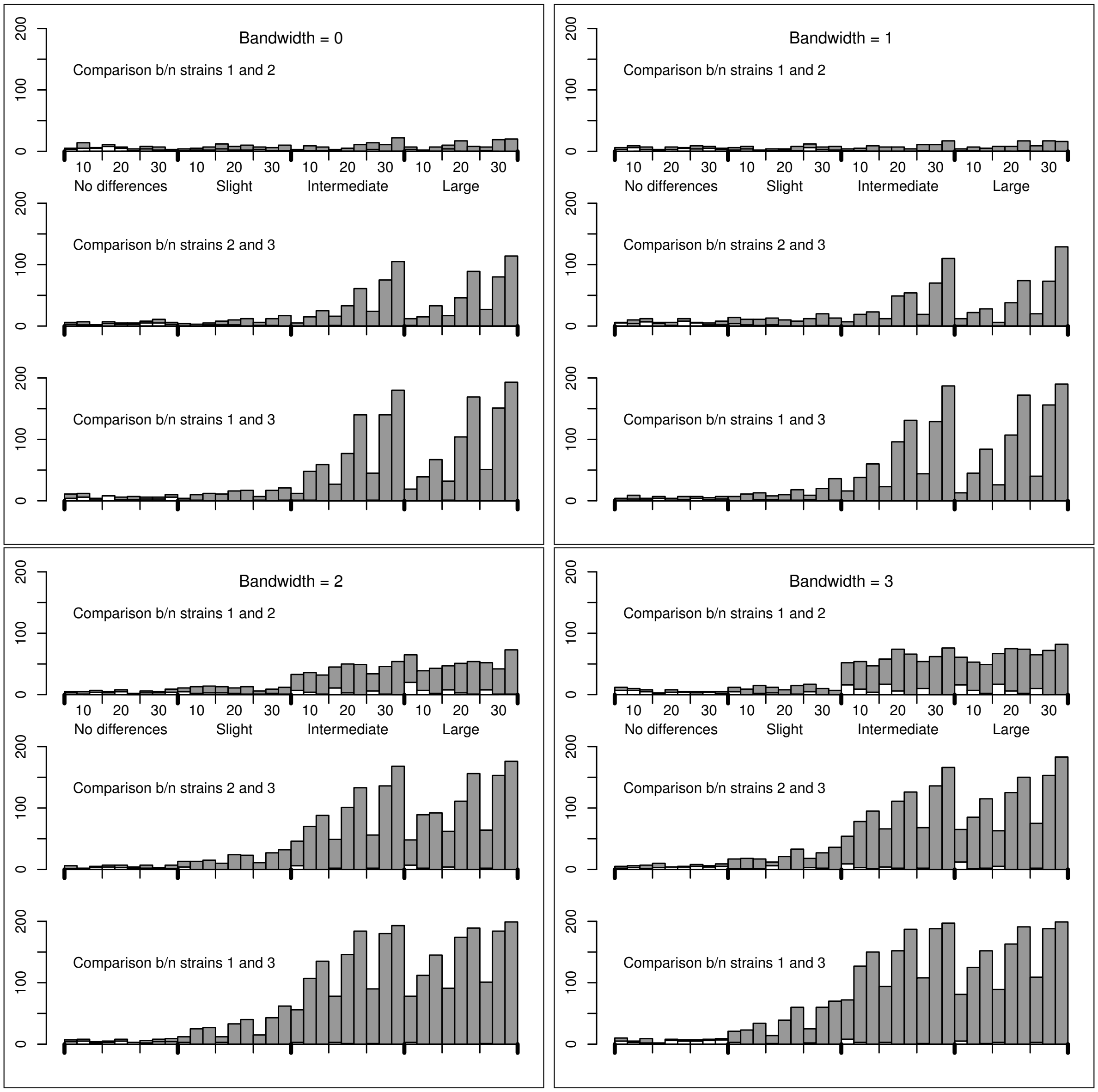

Figure S3: Numbers of test rejections for simulations performed under the regression models. Grey bars: number of times that the null hypothesis was rejected and that the alternative was true; White bars: number of times that the null hypothesis was rejected and that the alternative was wrong. The rejection threshold was fixed at 0.05/3 (using Bonferroni's correction). Between each consecutive ticks, there are three bars corresponding, from left to right, to 1, 5 and 10 collected samples per sampling site.

B Simulation under the mechanistic model and additional results

B.1 Mechanistic model

In the mechanistic simulation model, the epidemic spreads over a 10×10 square grid with inter-node distance equal to one ($I = 100$), and at discrete integer times $t = 1, 2, \ldots, T = 7$. The epidemic is the sum of $S = 3$ sub-epidemics corresponding to S strains. The S sub-epidemics are mutually independent.

Immigration. Strain s immigrates in the grid at one single time and eventually at several grid nodes.

- The immigration time T_s^{immigr} is randomly drawn in $\{1, \ldots, T\}$ with higher probabilities for earlier times. The probability that $T_s^{immigr} = t \in \{1, \ldots, T\}$ is $(T-t)^2 / \sum_{k=1}^{T} (T-k)^2$.
- The number of immigration nodes is drawn from a binomial distribution with size N^2 and with probability $\alpha_1 \in (0, 1]$ (we used $\alpha_1 = 0.05$). The immigration nodes are uniformly drawn in the grid; let $\mathcal{I}_s \subset \{1, \ldots, I\}$ denote the set of immigration nodes for strain s.
- At time T_s^{immigr}, the numbers of pathogen units $U_s(i, T_s^{immigr})$ of strain s at immigration nodes $i \in \mathcal{I}_s$ are independently drawn under a Poisson distribution with mean $\alpha_2 > 0$ (we used $\alpha_2 = 5$).

Propagation. At time $t \in \{T_s^{immigr} + 1, \ldots, T\}$, given the numbers of units $\{U_s(i, t-1) : i = 1, \ldots, I\}$ of strain s at the preceding time $t - 1$, the numbers of units of strain s in nodes $i \in \{1, \ldots, I\}$ are independently drawn under Poisson distributions with means $\lambda_s(i, t)$:

$$U_s(i,t) \mid \{U_s(i', t-1) : i' = 1, \ldots, I\} \sim \text{Poisson}\{\lambda_s(i,t)\}$$
$$\lambda_s(i,t) = \beta_s \sum_{i'=1}^{I} U_s(i', t-1) \exp\{-d(i,i')/\gamma\},$$

where $\beta_s > 0$ is proportional to the infection strength of a unit of strain s; $d(i, i')$ is the Euclidean distance between nodes i and i'; $\gamma > 0$ is the dispersal parameter. Thus, only the nodes i' where strain s is present contribute to the spread of this strain to nodes i, the contribution being larger when i' is close to i. We consider that strains differs in their fitness represented in this model by the parameter $\beta_s > 0$. The coefficients β_s in the mechanistic model are the counterparts of the coefficient $z(s)$ in the following regression model (Equation (2) in the main text):

$$Z_i = \left(\sum_{s=1}^{S} p_i(s) z(s) \right) + \eta_i.$$

We carried out 1,600 simulations of the mechanistic model; 800 with the dispersal parameter γ equal to 0.2, 800 with $\gamma = 0.5$ (longer dispersal distances). Among each series of 800 simulations, 200 were made with equal coefficients: $(\beta_1, \beta_2, \beta_3) = (2.0, 2.0, 2.0)$, 200 with slight differences in the coefficients: $(\beta_1, \beta_2, \beta_3) = (1.9, 2.0, 2.2)$, 200 with intermediate differences: $(\beta_1, \beta_2, \beta_3) = (1.5, 2.0, 3.0)$, and 200 with large differences: $(\beta_1, \beta_2, \beta_3) = (1.0, 2.0, 4.0)$.

Sampling. At the two final times $T - 1$ and T, the quantities of pathogen units are measured at every nodes. Thus, we know the quantities $\sum_{s=1}^{S} U_s(i, t)$ for all $i \in \{1, \ldots, I\}$ and for $t \in \{T - 1, T\}$. This observation allows us to compute for each node i the growth variable defined in Equation (1) in the main text and satisfying, under the mechanistic model,

$$Z_i = \log \left(\frac{1 + \sum_{s=1}^{S} U_s(i, T)}{1 + \sum_{s=1}^{S} U_s(i, T - 1)} \right).$$

At the final time T of the epidemic, J pathogen units are sampled and classified with respect to the strain.

- The number n of sampling sites is the minimum between a target number n_0 and the total number of nodes where the pathogen is present at time T. The sampling sites are uniformly drawn in the subset of the grid where the pathogen is present; let $\mathcal{I} \subset \{1, \ldots, I\}$ denote the set of sampling nodes.

- At each sampling site $i \in \mathcal{J}$, m_i pathogen units are sampled (uniform sampling without replacement) and classified. The number m_i is the minimum between a target number m_0 and the total number of units $\sum_{s=1}^{S} U_s(i, T)$ in node i at time T.

The number of samples is $J = \sum_{i \in \mathcal{J}} m_i \leq n_0 \times m_0$.
We used different target numbers of sampling sites ($n_0 \in \{10, 20, 30\}$) and different target numbers of samples per sampling site ($m_0 \in \{1, 5, 10\}$) to study the effect of the sampling effort.

B.2 Application to a simulation with equal coefficients and short distance dispersal

We considered one of the mechanistic simulations described in *Material and Methods* corresponding to $\beta_1 = \beta_2 = \beta_3 = 2$ (equal coefficients) and $\gamma = 0.2$ (short distance dispersal). We used 30 sampling sites and 10 samples per site. Strain proportions were estimated with $b = 1$. Figures S4–S5 and Table S3 show the simulation and provide the results. In this case, the ranking was not significant.

B.3 Application to a simulation with increasing coefficients and short distance dispersal

We considered one of the mechanistic simulations described in *Material and Methods* corresponding to $\beta_1 = 1.5$, $\beta_2 = 2$ and $\beta_3 = 3$ (equal coefficients) and $\gamma = 0.2$ (short

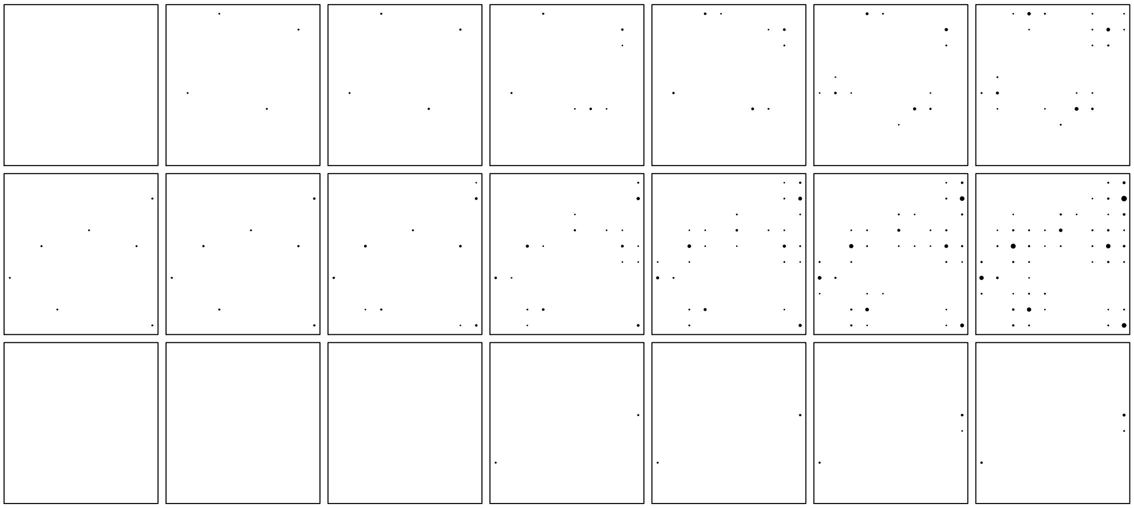

Figure S4: Spread of three strains of a pathogen simulated under the mechanistic model over a 10×10 square grid and over 7 time steps. The simulation was performed with equal coefficients ($\beta_1 = \beta_2 = \beta_3 = 2$) and short distance dispersal ($\gamma = 0.2$). Intensities of the three strains at time t are given by the t-th column of panels. Each row of panels provides the intensities of a given strain across time. Larger the dot, larger the intensity.

distance dispersal). We used 30 sampling sites and 10 samples per site. Strain proportions were estimated with $b = 1$. Figures S6–S7 and Table S4 show the simulation and provide the results. In this case, the ranking between strains 3 and 1 and between strains 3 and 2 was correct and significant. The ranking between strains 2 and 1 was correct but not significant.

B.4 Application to a simulation with increasing coefficients and long distance dispersal

We considered one of the mechanistic simulations described in *Material and Methods* corresponding to $\beta_1 = 1.5$, $\beta_2 = 2$ and $\beta_3 = 3$ (equal coefficients) and $\gamma = 0.5$ (long distance dispersal). We used 30 sampling sites and 10 samples per site. Strain proportions were estimated with $b = 1$. Figures S8–S9 and Table S5 show the simulation and provide the results. In this case, the ranking is not correct but is not significant.

Table S3: Results of the unilateral tests obtained for a simulation under the mechanistic model with equal coefficients ($\beta_1 = \beta_2 = \beta_3 = 2$) and short distance dispersal ($\gamma = 0.2$); the simulation is displayed in Figure S4.

Mechanistic coefficients	β_1	β_2	β_3
True value	2.00	2.00	2.00
Regression coefficient	$z(1)$	$z(2)$	$z(3)$
Estimated value	0.85	0.85	0.70
	$z(1) > z(2)$	$z(1) > z(3)$	$z(2) > z(3)$
p-value	0.487	0.336	0.337

Table S4: Results of the unilateral tests obtained for a simulation under the mechanistic model with increasing coefficients ($\beta_1 = 1.5$, $\beta_2 = 2$ and $\beta_3 = 3$) and short distance dispersal ($\gamma = 0.2$); the simulation is displayed in Figure S6.

Mechanistic coefficients	β_1	β_2	β_3
True value	1.50	2.00	3.00
Regression coefficient	$z(1)$	$z(2)$	$z(3)$
Estimated value	0.44	0.62	1.17
	$z(2) > z(1)$	$z(3) > z(1)$	$z(3) > z(2)$
p-value	0.282	0.002	0.002

Table S5: Results of the unilateral tests obtained for a simulation under the mechanistic model with increasing coefficients ($\beta_1 = 1.5$, $\beta_2 = 2$ and $\beta_3 = 3$) and long distance dispersal ($\gamma = 0.5$); the simulation is displayed in Figure S8.

Mechanistic coefficients	β_1	β_2	β_3
True value	1.50	2.00	3.00
Regression coefficient	$z(1)$	$z(2)$	$z(3)$
Estimated value	1.90	1.68	1.73
	$z(1) > z(2)$	$z(1) > z(3)$	$z(3) > z(2)$
p-value	0.393	0.406	0.464

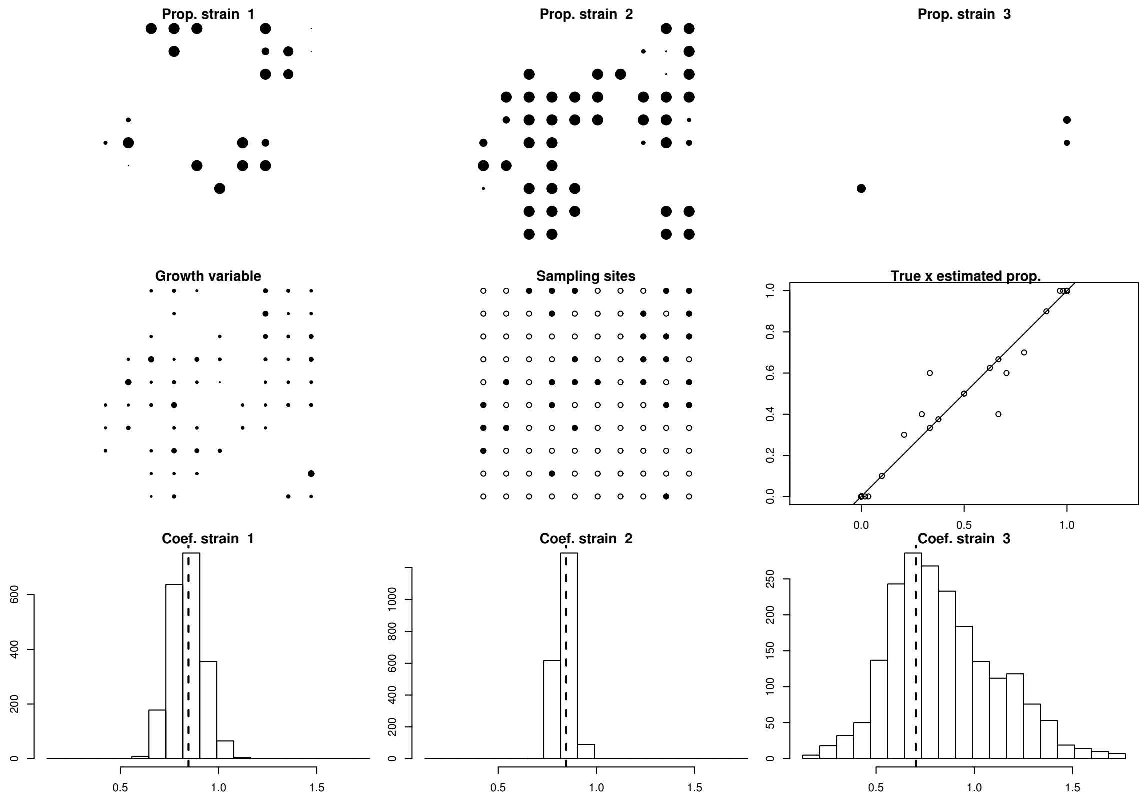

Figure S5: Data and results obtained for a simulation under the mechanistic model with equal coefficients ($\beta_1 = \beta_2 = \beta_3 = 2$) and short distance dispersal ($\gamma = 0.2$); the simulation is displayed in Figure S4. Top panels: Simulated proportions of each strain. Centre-left: Simulated values of Z_i (growth variable). Centre: Sampling sites (filled circles). Centre-right: True values of proportions of strains (abscissa) versus estimated values (ordinate). Bottom panels: Estimated values of the coefficients $z(s)$ (dashed lines), and corresponding permutation-based distributions under the null hypothesis of coefficient equality (histograms).

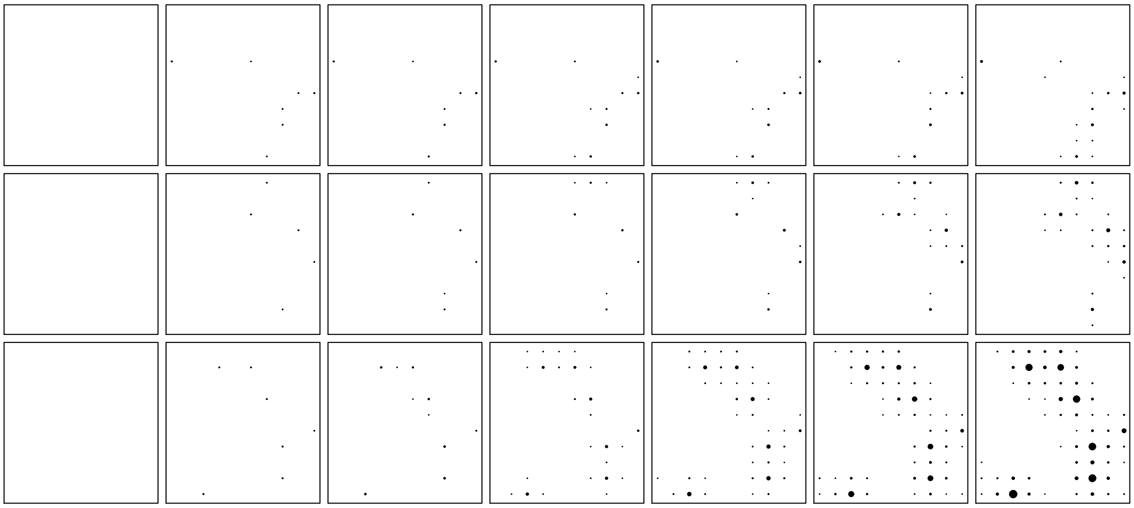

Figure S6: Spread of three strains of a pathogen simulated under the mechanistic model over a 10×10 square grid and over 7 time steps. The simulation was performed with increasing coefficients ($\beta_1 = 1.5$, $\beta_2 = 2$ and $\beta_3 = 3$) and short distance dispersal ($\gamma = 0.2$). Intensities of the three strains at time t are given by the t-th column of panels. Each row of panels provides the intensities of a given strain across time. Larger the dot, larger the intensity.

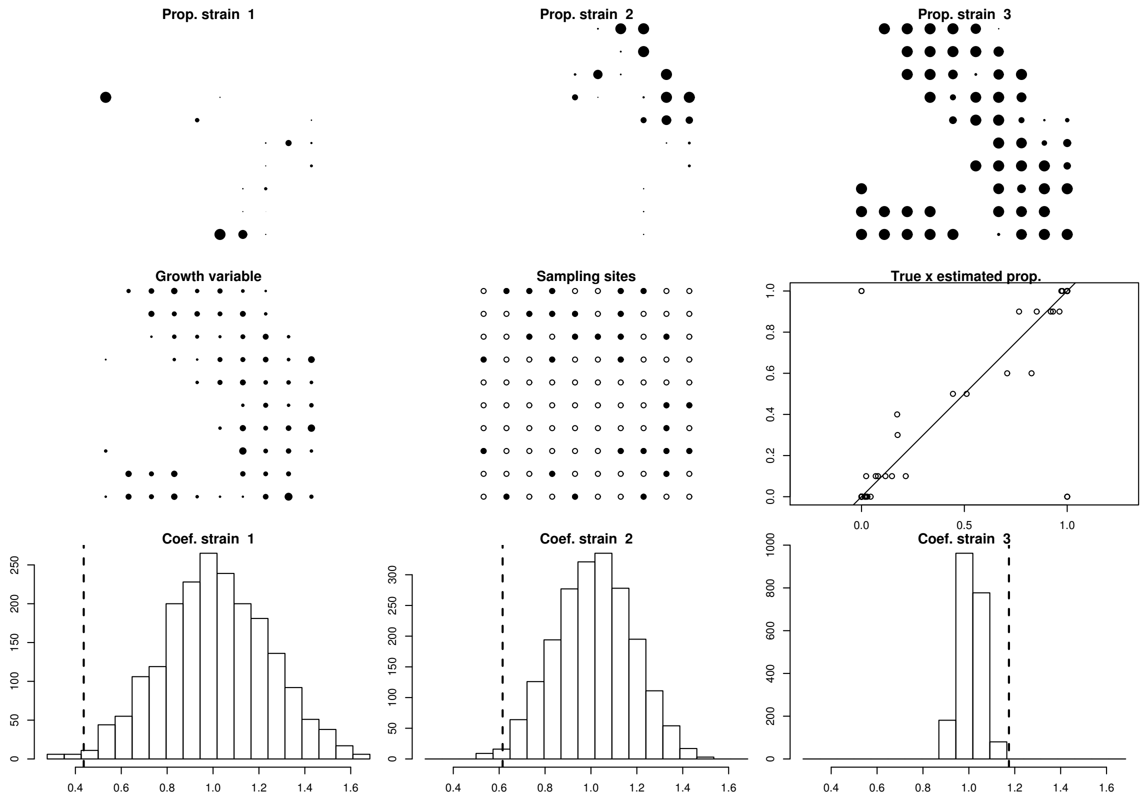

Figure S7: Data and results obtained for a simulation under the mechanistic model with increasing coefficients ($\beta_1 = 1.5$, $\beta_2 = 2$ and $\beta_3 = 3$) and short distance dispersal ($\gamma = 0.2$); the simulation is displayed in Figure S6. Top panels: Simulated proportions of each strain. Centre-left: Simulated values of Z_i (growth variable). Centre: Sampling sites (filled circles). Centre-right: True values of proportions of strains (abscissa) versus estimated values (ordinate). Bottom panels: Estimated values of the coefficients $z(s)$ (dashed lines), and corresponding permutation-based distributions under the null hypothesis of coefficient equality (histograms).

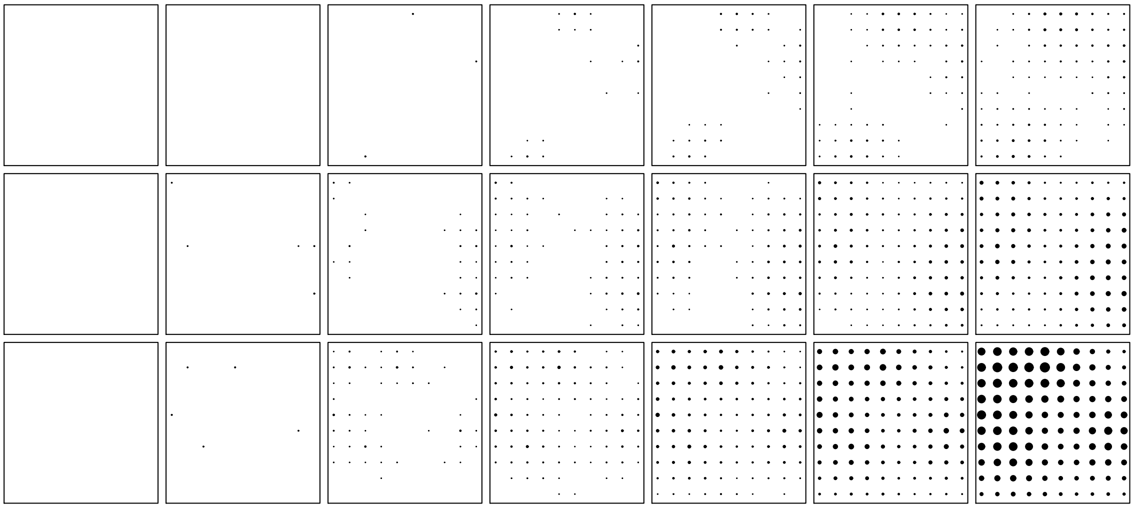

Figure S8: Spread of three strains of a pathogen simulated under the mechanistic model over a 10×10 square grid and over 7 time steps. The simulation was performed with increasing coefficients ($\beta_1 = 1.5$, $\beta_2 = 2$ and $\beta_3 = 3$) and long distance dispersal ($\gamma = 0.5$). Intensities of the three strains at time t are given by the t-th column of panels. Each row of panels provides the intensities of a given strain across time. Larger the dot, larger the intensity.

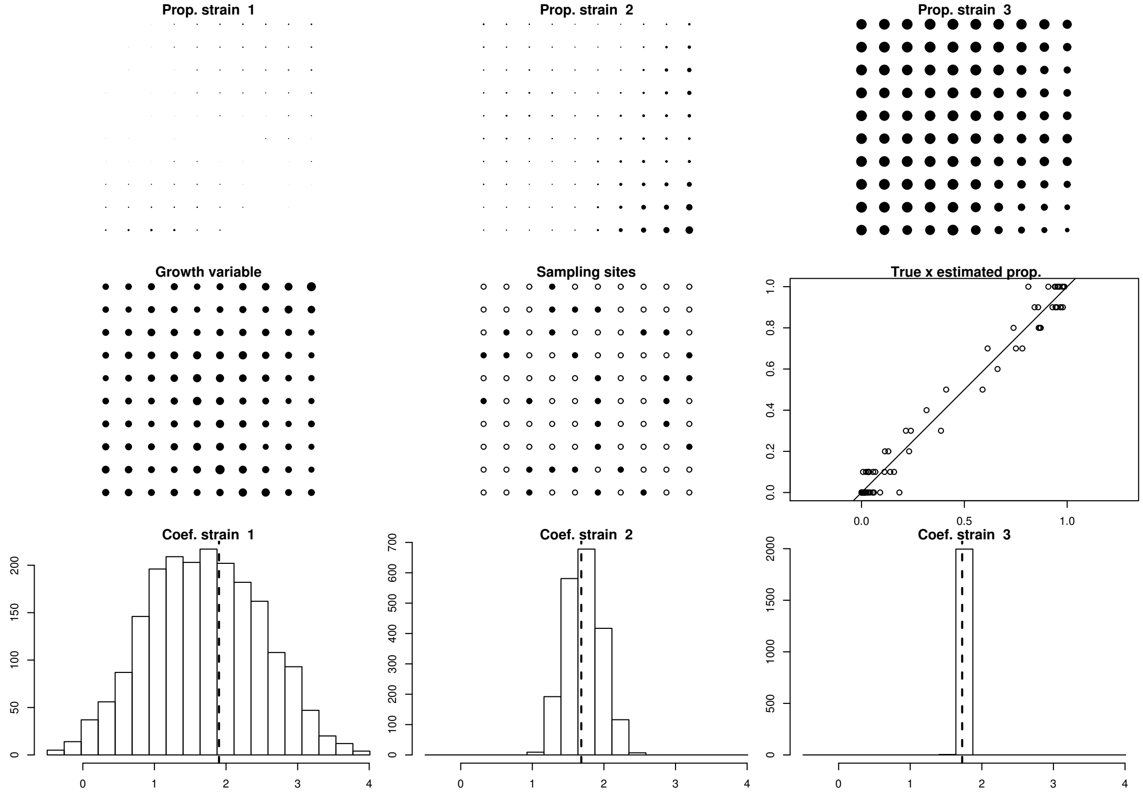

Figure S9: Data and results obtained for a simulation under the mechanistic model with increasing coefficients ($\beta_1 = 1.5$, $\beta_2 = 2$ and $\beta_3 = 3$) and long distance dispersal ($\gamma = 0.5$); the simulation is displayed in Figure S8. Top panels: Simulated proportions of each strain. Centre-left: Simulated values of Z_i (growth variable). Centre: Sampling sites (filled circles). Centre-right: True values of proportions of strains (abscissa) versus estimated values (ordinate). Bottom panels: Estimated values of the coefficients $z(s)$ (dashed lines), and corresponding permutation-based distributions under the null hypothesis of coefficient equality (histograms).
